# Effects of Empowerment Program on the Burden of Care in Mothers of Children with Phenylketonuria

**Published:** 2019

**Authors:** Abolfazl RAHGOI, Tahere SOJOODI, Masoud FALLAHI KHOSHKNAB, Mehdi RAHGOZAR, Soheila SHAHSHAHANI

**Affiliations:** 1 Nursing Department, University of Social Welfare and Rehabilitation Sciences, Tehran, Iran; 2 Department of Statistics, University of Social Welfare and Rehabilitation Sciences, Tehran, Iran; 3 Pediatric Neurorehabilitation Research Center, University of Social Welfare and Rehabilitation Sciences, Tehran, Iran

**Keywords:** Empowerment, Burden of care, Phenylketonuria (PKU)

## Abstract

**Objectives:**

Phenylketonuria (PKU) is a genetic disease of children that need a lifelong diet for its treatment. Because of the high burden of care, parents and particularly mothers are prone to mental disorders or psychiatric adverse reactions. We aimed to investigate the effect of empowerment of mothers on the burden of caring for children with PKU.

**Materials & Methods:**

This was a semi-experimental (before-after with control group) study. Overall, 50 mothers of children with PKU that were referred to Endocrine Clinic in Qods Hospital of Qazvin City, northwestern Iran in 2016, were selected and randomly divided into intervention and control groups. Data collection was made by using demographic characteristics of mothers and children and also the Zarit burden interview questionnaires. Empowerment program was delivered during nine sessions of holistic and family oriented empowerment model in the intervention group. The control group received routine services. Data were analyzed using descriptive and inferential statistics through SPSS software.

**Results:**

In intervention group, the mean score of overall care burden and its dimensions in the mothers of children with PKU in post-test was significantly lower than the pre-test (41.20 ± 5.04 vs. 58.24 ± 3.96; *P*<0.001); but pre-test and post-test scores in the control group had no significant difference (58.4 ± 5.22 vs. 58.96 ± 4; *P*=0.327). In other words empowerment program was able to reduce the burden of caring in mothers.

**Conclusion:**

Empowerment program can reduce the burden of care in mothers of children with PKU. Thus empowerment training is necessary for this group of mothers in the form of workshops and educational pamphlets.

## Introduction

Phenylketonuria (PKU) is a genetic disorder caused by a deficiency of the phenylalanine hydroxylase enzyme (1, 2). If the disease does not recognize early in life or the affected children with PKU do not be compliant with treatment regimen (food), it leads to severe cognitive or behavioral problems, seizures and autistic symptoms (3). The incidence of PKU is 1 case per 10000 live births (4). Despite studies that took place in recent years on families with PKU in Iran, due to the lack of a generalized screening and registering system, exact extent of the disease is unclear in Iran, however, the rate of PKU in Iran is about 1 in 6000 (5).

Caring for a child with an incurable disease has negative impact on parents. Parents of children with chronic diseases have vulnerable personality because they think that they are responsible for their child's condition or are unable to properly care for him. In some cases, the identity of the family may be disintegrated and parents may show psychological disorders (6). Similarly, parents of children with PKU may feel guilty for transmission of affected gen to their children, so they blame themselves (7). Caring for these patients, have a burden of care for parents. In most cases, control of phenylalanine level, maintaining accurate and balanced diet and handling, occupational and speech therapy programs are assigned to mothers (5). Because of the burden of care, many of them may show some degree of psychological reactions including emotional distress, confusion, anger, frustration, impatience, crying, obsession that leads to disturbed sleep and appetite, anxiety and depression (8, 9). "Burden of care is a concept emerging in the literature that describes the physical, emotional, social, and financial problems experienced by family caregivers" (10). Burden refers to the impact that induced from caring process of a sick person who is unable to perform his/her Activities of Daily Living (ADL). This impact affected the physical, financial, psychological, and social aspects of life of a caregiver (11). Care burden is a term frequently used for caregivers who provide informal care, that is, they do not have an educational background in the health field and are not paid for providing such care (11). Burden of care refers to the negative reactions that this person's experience (12). It occurs because of lack of balance between the needs and times spent on caring and other duties and also social, personal, physical and emotional roles of careers (13). Frequently, burden of care is more defined by its impacts and consequences on caregivers. It has both subjective and objective aspects. Subjective aspects refers to degree of impression that caregivers feel and its objective aspects show effects of burden on the family life like as performing of daily tasks (14).

Empowerment is a nursing intervention and educational model that helps mothers to feel the desired change (15, 16). “Empowerment paradigm involves a fundamental redefinition of roles and relationships of health care professionals and patients” (16). Family empowerment can lead to increase the life expectancy and increasing quality of life in patients with common genetic diseases such as thalassemia, hemophilia or PKU (17). Family-centered empowerment programs emphasize that family has an effective role on the motivation, psychological, knowledge, attitudes and perceived threat of the members and its primary goal is to empower family system that can lead to health promotion (18). So far family empowerment model is used in several studies on parents of children with chronic diseases such as asthma (6), ostomy (18), thalassemia (15), cognitive disabilities (19), psychological problems (20) and the results of this studies showed the positive impact of family empowerment model in improving the quality of life and health status of care providers. 

Due to limitations of these studies and the lack of local researches on patient with PKU, we aimed to evaluate the impact of family empowerment on the burden of caring in mothers of children with phenylketonuria in the Qazvin City in Iran.

## Materials & Methods

This was a semi-experimental study, before-after with control group and was performed on 50 mothers of children with PKU that referred to the Outpatient Clinic of Qods Hospital of Qazvin City north-west of Iran in 2016. 

Purposeful convenient sampling was used. The inclusion criteria were medical diagnosis of PKU in children, the age of the children ≤12 yr, living in Qazvin Province, caregiver willingness and cooperation to participate in the study. Exclusion criteria were parental refusal to cooperate with the research. By considering the 95% confidence interval, α=5% and 80% test power, the estimated sample size was 50 mothers that randomly assigned to intervention 25 and control 25 group. 

All participants were informed about the experimental process. The ethical protocol of this study was based on the Declaration of Helsinki and the obtained written informed consent signed by all participants, prior to their enrollment in the study. The study was approved by the Research Committee and thereafter by the Ethics Committee of the University of Social Welfare & Rehabilitation Sciences (license number: IR. USWR. REC. 1395.227) and Iranian Registry of Clinical Trials (ID: IRCT2016071828975N1). 

Data collection was made using maternal and children demographic questionnaires and also Zarit Burden of Care Index. Zarit questionnaire has 22 questions in 4 domains. It asks family caregivers about areas that may cause stress and strain such as physical (9 items), psychological (7 questions), economic (2 items) and relational problems (4 items). Scoring of answers is based on a five-point Likert scale ranging from 0 (never) to 4 (always). Scores are added to give total score that ranges from zero to 88, and earning a higher score indicates a greater burden of care (21, 22). The psychometric properties of the ZBI include acceptable inter-item reliability and convergent validity, indicated by a Cronbach's alpha of 0.79 and a correlation coefficient of 0.71, between caregiver's global evaluation and ZBI scores (21, 23). Test-Retest reliability of 0.71 and internal consistency (Cronbach's alpha=0.91) also have been reported (24). This questionnaire was translated into Persian and validated (25).

As pre-test, the questionnaire was completed by both groups. Then empowerment intervention program was done in nine 60-90 min sessions as twice a week by research team (Table 1). One month after the ending of intervention, both groups completed the questionnaire as post-test. For ethical purposes, at the end of the research, compacted training sessions were held for control group.

**Table 1:** Family-centered empowerment training program

first step	perceived susceptibility (2 sessions)	Explanations were offered about disease process, prognosis, symptoms, complications, risk factors and also consequences of not following the treatment program.
	perceived severity (1 sessions)	Plain language explanation about methods of prevention and control of PKU, correct behavior and their importance. Active participation of mothers in meeting sessions, discussion about subjects that were taught in the previous session.
second step	problem-solving methods (3 sessions)	Meeting was about self-efficacy promotion and includes practical difficulties of facing to problem, the process of problem solving, providing the solution for problem, encouraging to participate in choosing the best solution for the problem, analyzing the problem according to the causes and how it develops, providing goals of study and considering the solutions.
third step	Educational partnership (2 sessions)	In these sessions for improving mothers' Dignity (Sense of responsibility) by using promotion of self-esteem, self-Efficacy and self-control, they asked to have educational partnership for informing the other Family members. We gave them educational pamphlets and books about nutrition and dietary treatment of PKU to be studied by themselves and also educating their family members.
fourth step	Evaluation (1 session)	the evaluation was done in two stages, first one performed at the end of each session to ensure the learning of educated material that were presented in each session and the second one was performed one month after completing the empowerment program (by using Zarit burden of care)

Data were analyzed using SPSS version 18 (Chicago, IL, USA) by using descriptive statistics (frequency, percentage, mean and standard deviation) and chi-square tests, independent and paired t-test. In order to determine the effectiveness of the intervention, we used the t-test. The significant level of *P*<0.05 was considered. 

## Results

Sample was formed from 50 mothers of children with PKU. The average age of mothers in the intervention group (35.7 ± 5.6 yr) and control (36.1 ± 6.4 yr) and also other demographic characteristics in two groups were not significantly different (Table 2).

**Table 2:** Demographic characteristics of mothers of children with PKU

variable		Intervention N (%)	Control N (%)
Education	illiterate	0 (0)	2 (8)
	Elementary and guidance	14 (56)	17 (68)
	High school diploma	11 (44)	6 (24)
	University	0 (0)	0 (0)
Economic status	Poor	13 (52)	15 (60)
	Average	11 (44)	10 (40)
	Good	1 (4)	0 (0)
Gender of affected child	girl	12 (48)	14 (56)
	Boy	13 (52)	11 (44)

Before intervention, the mean and standard deviation of burden of care in the intervention and the control group were 58.24 ±3.96 and 58.96 ± 4 respectively and based on independent t-test, there was not any statistically significant difference in the burden of care between the two groups. After the intervention, the mean and standard deviation burden of care in the intervention and the control group were 41.20±25.04 and 58.40±5.22 and the difference between the two groups was statistically significant (*P*<0.001). Comparing the score of Zatite burden of care in intervention group before and after the intervention, the total and each dimension score of burden of care was decreased after the intervention and using dependent t-test showed that this decrease in scores was statistically significant (*P*<0.001). Total and each dimension score of burden of care of intervention group in pre-test were greater than post-test and this difference was statistically meaningful (*P*<0.001). In other words, the empowerment program decreased the burden of care in mothers of children with PKU (Table 3). 

**Table 3:** Burden of care in mothers of children with PKU before and after intervention

Dimensions	Group time	Intervention (n = 25)	Control (n =25)	p-value**
burden of care Total	Before intervention	58.24 ± 3.96	58.96 ± 4	0.526
	After intervention	41.20 ± 5.04	58.40 ± 5.22	0.0001
	p-value *	0.0001	0.327	-
psychological	Before intervention	18 ± 1.38	18.28 ± 1.72	0.529
	After intervention	13.72 ± 2.87	18.28 ± 1.72	0.0001
	p-value *	0.0001	-	
relational	Before intervention	9.92 ± 1.32	9.84 ± 1.49	0.842
	After intervention	6.60 ± 1.91	9.76 ± 1.58	0.0001
	p-value *	0.0001	0.327	
economic	Before intervention	5.68 ± 0.74	6 ± 0.1	0.05
	After intervention	3.44 ± 1.47	6 ± 0.92	0.0001
	p-value *	0.0001	0.337	
physical	Before intervention	24.64 ± 1.89	27 ± 24.84	0.663
	After intervention	17.44 ± 3.72	27 ± 24.44	0.0001
	p-value *	0.0001	0.393	

## Discussion

This study was performed to investigate the effect of maternal empowerment on the burden of caring in mothers of children with PKU. The findings showed that empowerment program lead to decrease the burden of care in mothers of children with PKU and our finding is compatible with another study done on mothers of children with cerebral palsy (26). In this type of empowerment, interaction between the medical team and the family creates a sense of control; improve the ability and skills in the family (27). The diversity and intensity of different caring roles can lead to psychological burden and stress in caregivers and especially to mothers of patients. This burden of care had a negative impact on their lives (25). The implementation of family-centered empowerment model was improved the quality of life of parents of children with chronic diseases (6). Using the family empowerment program lead to increment of maternal capability and improving their quality of life (15). 

A study examined the effect of educational program on the burden of care of family of hemodialysis patient. Educational programs were able to reduce the burden of family caregiving (28). 

The effect of educational program on reducing the burden of care was shown in family of patients with mental disorders (25). Family empowerment programs lead to increase of knowledge and attitudes of mothers of children with thalassemia (29). Despite the diversity of target groups in these studies, in general, family empowerment programs by improving the quality of life and increasing the knowledge and attitude of caregivers can reduce the burden of caring.

This study had some limitations, one of the most important of them is that only mothers participated in our educational program and we cannot evaluate the impact of empowerment program on reducing the burden of care in fathers. Because of lack of families' cooperation, we were not able to evaluate the long-term effect of empowerment programs. Another limitation was the lack of similar regional or international studies on this group of families to compare the findings with them. 

**Inconclusion, **By considering the positive effect of family empowerment program on reducing the burden of care in mothers of children with PKU, health service providers consider such suitable programs to support the families.
